# The Silent Threat: Unveiling Pathogenicity and Antimicrobial Resistance in Nonfermenting Gram-Negative Bacilli

**DOI:** 10.7759/cureus.108761

**Published:** 2026-05-13

**Authors:** Dhanashree R Chavan, Ravindra V Shinde, Satish R Patil

**Affiliations:** 1 Department of Microbiology, Krishna Institute of Medical Sciences, Krishna Vishwa Vidyapeeth (Deemed to Be University), Karad, IND

**Keywords:** acinetobacter baumannii (a. baumannii), antimicrobial resistance, burkholderia species, nonfermenting gram-negative bacilli (nfgnb), pathogenicity, pseudomonas aeruginosa (p. aeruginosa), stenotrophomonas maltophilia (s. maltophilia)

## Abstract

Nonfermenting Gram-negative bacilli (NFGNB) are aerobic bacteria prevalent in healthcare settings and have become important healthcare-associated pathogens due to their capacity to persist in hospital environments and their intrinsic and acquired antibiotic resistance. Due to their significant involvement in multidrug-resistant infections, the World Health Organization has assigned carbapenem-resistant *Acinetobacter baumannii* and *Pseudomonas aeruginosa* as priority pathogens. The excessive administration of broad-spectrum antibiotics, notably carbapenems, has driven the global increase in carbapenem-resistant NFGNB, resulting in limited options for treatment and increasing morbidity and mortality. NFGNB, including *P. aeruginosa*, *A. baumannii*, *Stenotrophomonas maltophilia*, and Burkholderia species, are significant opportunistic pathogens associated with serious nosocomial infections, particularly in critically ill and immunocompromised individuals. Their increasing resistance contributes significantly to the worldwide antimicrobial resistance crisis, which is currently acknowledged as a serious public health issue.

## Introduction and background

The taxonomically broad group of aerobic, nonsporing bacilli known as nonfermenting Gram-negative bacilli (NFGNB) either do not use glucose as an energy source or use it oxidatively [1]. They exist in the environment as saprophytes, and some can also be found in the human intestine as commensals. NFGNB constitute approximately 15% of all bacterial isolates obtained in clinical microbiology laboratories. They have been implicated in a range of infections, including septicemia, meningitis, pneumonia, urinary tract infections, and surgical site infections [2]. The pathogenic role of NFGNB is well documented, particularly among patients currently or recently hospitalized. Additional risk factors for NFGNB infections include immunocompromised individuals (such as oncology and organ transplant patients), those with cystic fibrosis, trauma patients, individuals receiving mechanical ventilation, and patients with indwelling urinary catheters. The priority pathogen list was released by the World Health Organization (WHO) to address the rise in antibiotic resistance worldwide. In this list, the risk associated with Gram-negative bacilli, which are resistant to several antibiotics, is underlined. Two NFGNB species, namely *Acinetobacter baumannii *and *Pseudomonas aeruginosa* (carbapenem-resistant), are some of the species in this list [3].

NFGNB exhibit intrinsic resistance to multiple antibiotics and are known to produce extended-spectrum β-lactamases (ESBLs) and metallo-β-lactamases. Nonfermenters have developed resistance to many commonly used antibiotics, including cephalosporins and carbapenems. This resistance limits treatment options, leading to prolonged hospital stays, increased mortality, and higher healthcare costs. Pseudomonas species are the most commonly isolated NFGNB from clinical specimens, followed by Acinetobacter species, Burkholderia species, and *Stenotrophomonas maltophilia* (SM), among others [2,4]. This narrative review provides a comprehensive overview of NFGNB, emphasizing their epidemiology, clinical manifestations, and mechanisms of antimicrobial resistance (AMR).

## Review

Methodology

Search Strategy

**Figure 1:**
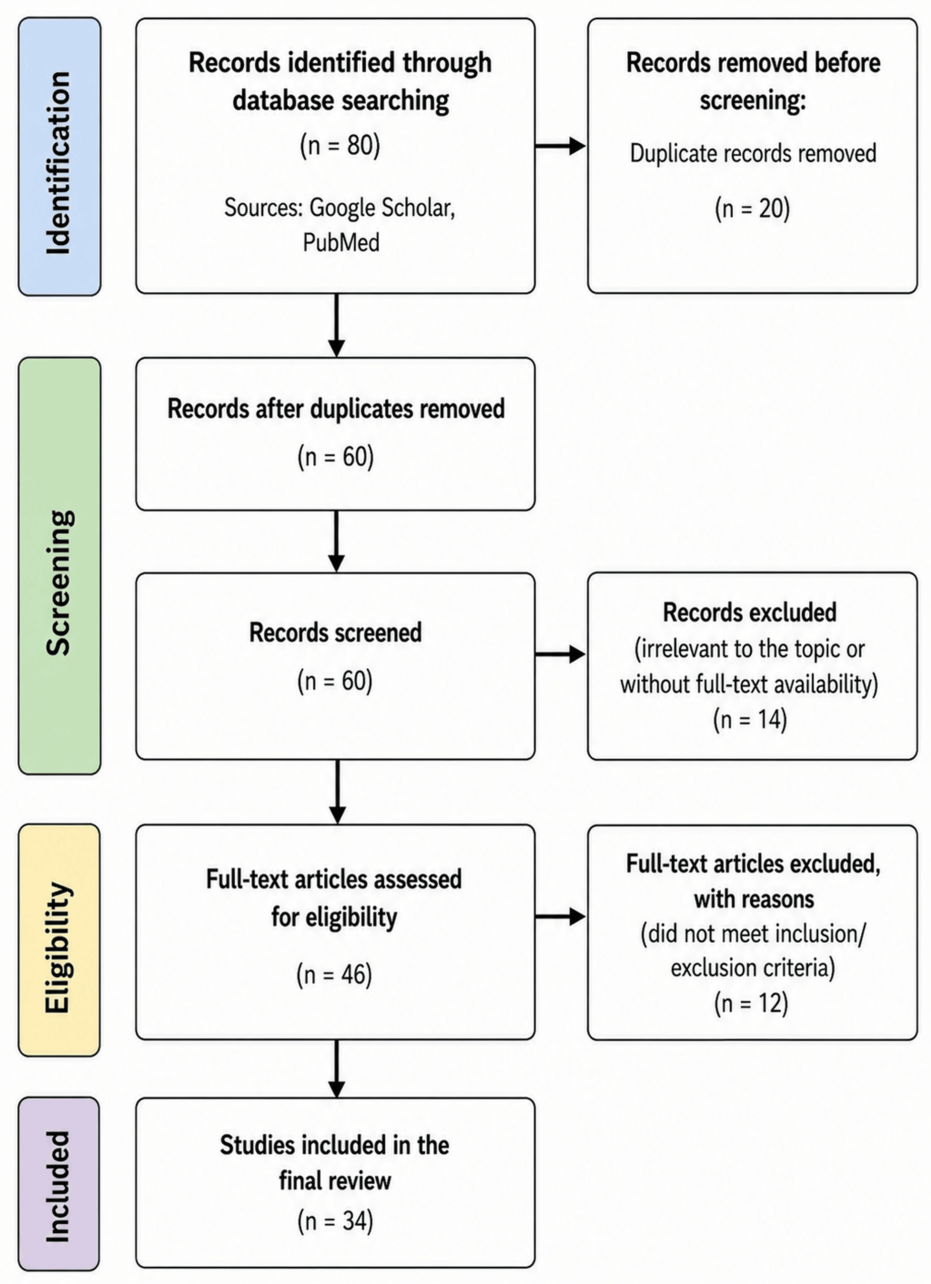
Article selection and screening process Image credit: This is an original image created by the authors based on a literature search strategy

A thorough literature search using databases such as Google Scholar and PubMed served as the basis for creating this review article on NFGNB. The search included publications that utilized Medical Subject Headings terms such as “NFGNB infections”, “infections of Pseudomonas”, “pathogenicity and resistance mechanism of Pseudomonas”, “infections of Acinetobacter”, “pathogenicity and resistance mechanism of Acinetobacter”, “infections of Stenotrophomonas”, “pathogenicity and resistance mechanism of Stenotrophomonas”, “infections of Burkholderia”, “pathogenicity and resistance mechanism of Burkholderia”. Studies were screened for relevance, and those unrelated to the topic were excluded. Publications without full-text availability were also excluded. The initial search yielded 80 records related to NFGNB. After removing duplicate records, 60 records remained and were screened based on titles and abstracts. During this stage, 14 records were excluded for being irrelevant to the topic or lacking full-text availability. Subsequently, 46 full-text articles were assessed for eligibility, of which 12 were excluded for not meeting the inclusion criteria. Finally, 34 studies were included in the review. A schematic of the literature selection process is shown in Figure 1.

Inclusion Criteria

Only English-language published articles were included. Original research articles and review articles reporting on NFGNB, including their identification, associated infections, and resistance mechanisms, were considered eligible.

Exclusion Criteria

Articles with insufficient data were excluded. Studies not related to NFGNB or lacking relevant data on identification or resistance mechanisms were also excluded. Duplicate articles were excluded.

Brief scenario

NFGNB are widely distributed and can grow in various healthcare environments, including sinks, parenteral fluids, medical equipment, and distilled water, owing to their inherent resistance to frequently used disinfectants [4]. Over recent years, however, NFGNB has emerged as a major cause of nosocomial infections because it can persist in hospital environments and has intrinsic antibiotic resistance [5]. Improper or excessive use of broad-spectrum antimicrobials such as carbapenems promotes the emergence of carbapenem-resistant bacteria. Resistance of Gram-negative bacteria to carbapenems has emerged as a worldwide issue. There are fewer therapeutic options available to treat certain infectious diseases as a result of rising AMR and limited development of new antibiotics. Empirical therapy for nosocomial and healthcare-associated infections should include antibiotics effective against *P. aeruginosa*. However, empirical treatment targeting Acinetobacter spp. and SM is not routinely recommended in critically ill patients, as it may delay appropriate therapy. Therefore, the rise of multidrug-resistant (MDR) bacteria often requires combination or broad-spectrum antibiotic therapy, particularly in nosocomial infections and severe sepsis or shock [6]. Carbapenems were once considered the last-resort treatment for NFGNB infections, but more recently, carbapenem-resistant NFGNB have been increasingly reported worldwide. These bacteria are often resistant to multiple classes of antimicrobial agents due to their various intrinsic and acquired resistance mechanisms, leaving few treatment options [7].

The WHO has stated that AMR is one of the top 10 threats to human health worldwide, necessitating immediate action to meet sustainable development objectives. In 2019, AMR caused 1.27 million deaths globally and resulted in millions of cases of prolonged illness and hospitalization. Global deaths could reach 10 million annually by 2050 if proper action against the spread of AMR is not immediately implemented. India has been described as the global capital of AMR. *Escherichia coli*, *Staphylococcus aureus*, *Klebsiella pneumoniae*, *Streptococcus pneumoniae, A. baumannii, *and *P. aeruginosa* are responsible for about 71% of all AMR-related pathogen deaths worldwide [8]. This review aims to provide a comprehensive understanding of NFGNB infections, with a focus on their resistance mechanisms.

Epidemiology and clinical significance

The environment is the habitat of nonfermenting Gram-negative bacteria, which are present in soil, water, plants, and foods such as dairy, poultry, and frozen meals, as well as on various surfaces. They may be isolated from medical apparatus like humidifiers, ventilation systems, dialysis and saline solutions, disinfectant substances, and medications, in addition to healthcare workers' skin and possess the ability to spread horizontally. They pose a challenge because they are resistant to several disinfectants commonly used in hospital settings. The most frequent microorganisms in the NFGNB group are Pseudomonas, Acinetobacter, Stenotrophomonas, Burkholderia, Alcaligenes, Weeksella, Flavimonas, Achromobacter, Ralstonia, Roseomonas, Sphingobacterium, Elizabethkingia, etc. [9]. Recently, because of the liberal and empirical use of antibiotics, NFGNB have become known as a major healthcare-associated infection. Currently, the most often isolated nonfermenters that are harmful to humans are *P. aeruginosa* and *A. baumannii *[10]. *P. aeruginosa* is a well-known opportunistic pathogen. The occurrence of MDR strains of this bacterium species is concerning. Because of their widespread and developing mechanisms of antibiotic resistance, these microorganisms are connected to considerable morbidity and mortality and are still challenging to treat; 10% of nosocomial infections are caused by them. *P. aeruginosa* is able to produce both acute and long-term infections [11-13].

Acinetobacter species are saprophytic and widely distributed, and have become significant nosocomial pathogens because they can survive on a wide range of dry and wet surfaces in hospital settings [14]. Acinetobacter infections, which were once thought to be commensal opportunists with minimal virulence and little clinical importance, have become more common and severe over the past few decades. This increase has been associated with the increased use of broad-spectrum antimicrobial therapy, central venous catheterization, and mechanical ventilation. Currently, infections caused by Acinetobacter species, particularly *A. baumannii,* are common in healthcare facilities, with the highest incidence in intensive care units (ICUs), which account for at least 20% of infections acquired in these hospital wards [15]. Although Acinetobacterspecies are frequently found in the environment, the primary source of *A. baumannii *and closely related pathogenic strains seems to be associated with humans. The most frequent pathogen among the species of Acinetobacter is *A. baumannii*. It poses a risk to public health since MDR and extensively drug-resistant strains are more prevalent [16]. The increasing frequency of carbapenem-resistant *A. baumannii *(CRAB) has exacerbated this issue, making it a critical public health and therapeutic priority. Carbapenems, somewhat regarded as antibiotics of last option, are ineffective against CRAB, leading to much greater fatality rates, ranging from 35% to 60% compared with infections caused by nonresistant strains [17].

SM* *is a globally distributed bacterium frequently isolated from environmental sources, mainly from water, soil, sediment, plants, and animal specimens. SM is an opportunistic bacterium intrinsically resistant to several commonly used broad-spectrum antibiotics [18]. SM is a Gram-negative MDR organism that is increasingly reported worldwide and is most frequently associated with respiratory infections in humans. In humans, it can result in a variety of serious illnesses. Hospital-acquired SM infections appear to be more prevalent, especially among immunocompromised patients. Furthermore, there have been documented cases of community-acquired SM infections. Both children and adults can develop SM infections [19]. According to standards for healthcare-associated infection surveillance published by the National Healthcare Safety Network, which is operated by the Centers for Disease Control and Prevention, the incidence of SM infections has been reported to range from 13% to 60% [20].

There are about 35 species with recognized names in the genus Burkholderia. While many Burkholderia species are recognized as phytopathogens, others have been reported to cause opportunistic infections in humans and animals. In particular, *Burkholderia pseudomallei *and *Burkholderia mallei *are important pathogens affecting both humans and animals, as they are resistant to a wide range of antimicrobial agents. The *Burkholderia cepacia* complex (Bcc) is recognized as a significant opportunistic pathogen in humans [21]. Bcc and *B. pseudomallei* complex bacteria have been reported to cause serious illnesses in humans and animals in recent years, posing significant challenges for both patients and healthcare providers [22].

Infection caused by NFGNB

Infection Caused by P. aeruginosa

*P. aeruginosa *is an opportunistic organism that causes a number of illnesses in humans. It is an opportunistic bacterium associated with illnesses in healthcare facilities, including ventilator-associated pneumonia, infections in intensive care units (ICUs), central line-related bloodstream infections, surgical site infections, urinary tract infections, burn wound infections, keratitis, and otitis media [23]. Infections caused by Pseudomonas are summarized in Table 1.

**Table 1:** Infection caused by Pseudomonas aeruginosa CF: cystic fibrosis; COPD: chronic obstructive pulmonary disease; ICU: intensive care unit; LPS: lipopolysaccharide

Infection	Risk factor	Mechanism
Respiratory tract infections		
Ventilator-associated pneumonia [24]	Mechanical ventilation >48 hours, prior antibiotic exposure, airway colonization, ventilation >5 days	Tracheobronchial colonization, transmission via medical equipment/cross, contamination, complex drug, resistance genes (plasmids/genome), biofilm formation
CF [24]	Hereditary disease (CF), hyperinflammatory lung environment	Biofilm formation, downregulation of virulence factors, upregulation of exopolysaccharides, evasion of immune recognition, and neutrophil attacks
COPD [25]	Smoking, prolonged exposure to air irritants	Progressive cough and mucus production
Bronchiectasis [26]	Patients with CF, other types of bronchiectasis, and severe COPD	Chronic bronchial dilatation → insufficient mucus drainage
Soft tissue and skin infections		
Burn wound infections [25]	Skin graft sites, extensive burns (>40% body surface), length of hospitalization, immunocompromised, or underlying pulmonary disorders	Initial colonization by *S. aureus* (skin microflora), secondary colonization by *P. aeruginosa* within 7 days post, injury
Skin and fascial layer infection [26]	Elderly patients, impaired immune systems	Rapid inflammation and tissue damage
Necrotizing fasciitis [26]	Elderly, immunocompromised individuals	Infection of subcutaneous tissue and fascia, propagation along the fascial plane correlated with subcutaneous thickness
Green nail syndrome/chromonychia/Fox-Goldman syndrome [26]	Onycholysis, onychotillomania, chronic paronychia, microtrauma to proximal nail fold, nail disorders (psoriasis), diabetes mellitus, immunosuppression, frequent exposure to water or moist conditions	Persistence of pyocyanin pigment in nail plate, *P. aeruginosa* colonization of nail bed, autologous spread by touching/scratching damaged skin
Ear infections		
Otitis externa (Swimmer’s ear) [26]	Prolonged exposure to wetness, insertion of foreign items, contaminated water (swimming pools, hot tubs)	Colonization of the external auditory canal
Chronic otomastoiditis/chronic tympanomastoiditis/active mucosal otitis media [26]	Persistent infection with *P. aeruginosa*	Long-term inflammation of the mastoid cavity and the middle ear
Urinary tract infections		
Catheter-associated urinary tract infection [24]	Indwelling urinary catheters, ICU patients, and hospitalized elderly populations	Catheter insertion disturbs mucosal epithelial layers → promotes colonization, exploits catheters as entry tool, biofilm formation on catheter surfaces, virulence factors: alginate, LPS, flagella, pili, proteases, elastases, toxins, hemolysins
Bloodstream infections		
Bacteremia [24]	Respiratory tract infections, central venous catheters, immunocompromised patients (especially ICU), underlying conditions (lung cancer), previous antimicrobial therapy	Type III secretion-dependent exo-products, quorum-sensing-dependent virulence
Infective endocarditis [27]	Intravenous drug abuse	Use of contaminated water or equipment during drug abuse
Joint and bone infections		
Periprosthetic joint infection [26]	Total knee arthroplasty (the most prevalent cause of revision), total hip arthroplasty (the third most common cause)	Germs enter sterile bones/joints via exogenous/endogenous contiguous foci or hematogenous spread
Osteomyelitis [26]	General susceptibility in bone infections	Opportunistic colonization of bone tissue
Sternoclavicular septic arthritis [26]	Intravenous drug use, diabetes mellitus, trauma, and infected central venous lines	Bacterial invasion of the sternoclavicular joint
Others infection		
Bacterial keratitis [26]	Contact lens wear (especially prolonged), lens contamination, eye surgery, ocular diseases	Damage to corneal epithelial surface → increased risk of abrasions, opportunistic infection when epithelial barrier is compromised, binding to toll-like receptor 5 on cornea → rapid internalization

Infection Caused by A. baumannii

*A. baumannii* presents a serious concern in healthcare settings because of its ability to become resistant to a wide range of antimicrobial agents. It is known to cause serious and invasive nosocomial infections, including ventilator-associated pneumonia, bloodstream infections, urinary tract infections, soft-tissue and skin infections, and meningitis. *A. baumannii* can cause infections affecting multiple organ systems, ranging from mild to severe [28,29]. Infections caused by Acinetobacter are shown in Table 2.

**Table 2:** Infection caused by Acinetobacter baumannii COPD: chronic obstructive pulmonary disease; ICU: intensive care unit Source: [29]

Infection	Risk factor	Mechanism
Hospital-acquired pneumonia	Hospitalization ≥48 hours, mechanical ventilation, underlying conditions (diabetes, COPD), immunocompromised, elderly, antibiotic-treated patients	Spread via person-to-person transmission, survival on abiotic surfaces
Bacteremia	Hospitalized patients, weakened immune systems, life support (ventilation, catheters), underlying conditions (diabetes, COPD), elderly, immunocompromised, prolonged antibiotic consumption	Bacteria enter the bloodstream and circulate, enzyme production neutralizes antibiotics, the development of biofilms protects microorganisms from antibiotics, and host immunity
Urinary tract infection	Women (more likely than men), long ICU stay, life support (ventilation, catheters), underlying conditions (diabetes, COPD)	Bacterial colonization and antibiotic resistance complicate therapy
Wound infection	Compromised immune systems, underlying conditions (diabetes, COPD), elderly patients, history of antibiotic use, battlefields, and high-risk environments	Bacteria enter the wound → inflammation and tissue damage, antibiotic resistance complicates therapy
Meningitis	Infection of the protective membranes of the brain and spinal cord	Bacterial invasion of the meninges

Infection Caused by SM

A wide range of clinical manifestations is associated with SM [30].* *Various infections caused by Stenotrophomonas are listed in Table 3.

**Table 3:** Infection caused by Stenotrophomonas maltophilia COPD: chronic obstructive pulmonary disease Source: [30]

Infection	Risk factor	Mechanism
Bacteremia	Presence of intravascular equipment (especially central venous catheters), environmental reservoirs (contaminated hemodialyzers, insufficient disinfection of capillary dialyzers)	Transmission from contaminated medical equipment (dialyzers, pressure monitoring systems)
Endocarditis	Intravenous drug abuse, surgery for prosthetic valves (early postoperative period)	Contamination introduced via vascular devices or anticoagulant reflux, complications include valve ring abscesses, myocardial abscesses
Nosocomial pneumonia	Mechanical ventilation, tracheostomy, prior use of broad-spectrum antibiotics, preexisting lung conditions (COPD, bronchiectasis, kyphoscoliosis, endobronchial obstruction), lung transplantation	Contamination from respiratory equipment (ventilator circuits, oxygen analyzers, reusable ventilator probes), inadequate sterilization of the bronchoscope, leading to isolation (without overt infection)
Community-acquired pneumonia (rare)	Predisposing conditions (rheumatic heart disease, bronchiectasis)	-
meningitis (uncommon)	Neonates and infants: spontaneous onset, adults: neurosurgical procedures, spinal epidural catheter (predisposing to spinal epidural abscess)	-
Ocular infections	Contact lens wearers, cataract extraction	-
Urinary tract infection	Hospital-acquired, urinary tract surgery, including catheterization, and disinfectant agent employed for bladder instillation, which contaminated	Infection usually acquired in a hospital setting; introduction via surgery, instrumentation, or contaminated disinfectant
Soft-tissue and skin infections	Accidental injury (including workplace accidents), surgical trauma, breach of cutaneous defenses at tracheostomy, suprapubic, and vascular catheter sites	Colonization vs. true infection often difficult to distinguish, and infection following trauma or an iatrogenic breach of skin barriers
Bone and joint infection (uncommon)	Orthopedic surgery, trauma, and abuse of intravenous drugs	-

Infection Caused by Burkholderia Species

Infections caused by Burkholderia species are challenging to treat because most pathogenic strains are naturally resistant to several classes of antibiotics [31]. Various infections associated with Burkholderia species are presented in Table 4.

**Table 4:** Infection caused by Burkholderia species Bpm: Burkholderia pseudomallei; CF: cystic fibrosis; CGD: chronic granulomatous disease Source: [31]

Burkholderia species	Infections	Risk factors	Mechanism
Bpm	Pneumonia (pulmonary melioidosis); localized skin lesions, arthritis, osteomyelitis, musculoskeletal melioidosis; neurological melioidosis; systemic infection/sepsis (acute, recurrent, chronic, or asymptomatic) if localized infection is not resolved	Diabetes mellitus, hazardous alcohol use, chronic lung disease, chronic renal disease, thalassemia, occupational/environmental exposure, immunodeficiency (e.g., cystic fibrosis)	Inhalation (most severe disease), skin inoculation (cutaneous lesions, which can progress to pulmonary disease), ingestion, translocation from the exposure site to other organs
Burkholderia cepacia complex	Acute pneumonia → recurrent/chronic pneumonia, osteomyelitis, meningitis, severe “cepacia syndrome” (necrotizing pneumonia + bacteremia → early death)	Immunodeficiency (especially cystic fibrosis, chronic granulomatous disease)	Inhalation of bacteria from the environment, progression from acute to recurrent/chronic or latent infection
Chronic infections			
Bpm	Chronic melioidosis (>2 months of symptomatic infection), localized and/or systemic manifestations, misdiagnosis as tuberculosis	More common during wet/monsoon seasons, occupational exposure (e.g., rice farmers in flooded paddies), CF	In Murine models - inhalation (pulmonary exposure), intravenous (systemic disease), intranasal (pulmonary model)
Burkholderia cepacia complex	Chronic pneumonia (most common), recurrent infections in CGD patients	CF, chronic granulomatous disease	-

Resistance mechanism

*P. aeruginosa* possesses multiple resistance mechanisms, which are usually categorized into three types: intrinsic, acquired, and adaptive [27].

Intrinsic Resistance

Resistance arises from the expression of antibiotic-expelling efflux pumps in the cell, the formation of enzymes that inactivate or hydrolyze antibiotics (e.g., ESBLs, AmpC beta-lactamases, or carbapenemases), and the poor permeability of its outer membrane. Downregulation of the outer membrane protein OprD is particularly important, which reduces permeability of the membrane to some antimicrobials and usually leads to carbapenem resistance. Chromosomal AmpC beta-lactamases are inducibly expressed and play a major role in the intrinsic resistance of *P. aeruginosa* to most penicillins and cephalosporins. Reduced sensitivity to beta-lactams is mainly caused by constitutive production of the MexAB-OprM efflux pump, whereas reduced susceptibility to aminoglycosides is somewhat caused by inducible expression of the MexXY efflux pump [32].

Acquired Resistance

Resistance can arise through horizontal gene transfer or mutational alterations. These can provide advantages such as reduced permeability, alterations in the drug target, overproduction of efflux pumps, or the production of enzymes that inhibit antibiotics. Common resistant Pseudomonas strains, such as those with ESBLs and carbapenem resistance, result from the accumulation of acquired mechanisms [27,32].

Adaptive Resistance

Through a variety of strategies, bacteria develop adaptive resistance to increase antibiotic resistance by temporarily altering the expression of genes and/or proteins in reaction to different environmental stimuli. In *P. aeruginosa*, the synthesis of biofilms is the most common mechanism for increasing adaptive antibiotic resistance. To increase its virulence, *P. aeruginosa* can form biofilms. Furthermore, the bacteria can also adapt to antibiotic therapy by generating persister cells, or persisters, reducing metabolic activity and limiting antibiotic targets. Persister cells from the persisters can preserve vitality and repopulate biofilms; once antibiotics are not available, bacteria will resume development and cause chronic illnesses [33]. Classification of *P. aeruginosa *based on antibiotic resistance is shown in Table 5 [32].

**Table 5:** Classification of P. aeruginosa based on antibiotic resistance (combining Magiorakos’s and Kadri’s definitions)

Type	Definition
Multidrug-resistant *P. aeruginosa*	Resistant to three or more types of antimicrobial agents that are effective against *P. aeruginosa*
Extensively drug-resistant *P. aeruginosa*	Resistant to at least one compound in every antimicrobial class, with the exception of two or fewer
Pan-drug-resistant *P. aeruginosa*	Resistant to any antibiotic agent
Difficult-to-treat *P. aeruginosa*	Resistant to piperacillin-tazobactam, ceftazidime, cefepime, aztreonam, meropenem, imipenem-cilastatin, ciprofloxacin, and levofloxacin

*A. baumannii:*Due to Acinetobacter’s complex and diverse resistance mechanisms, major groups of antibiotics are at risk of losing their efficacy against this bacterium with the increasing incidence of Acinetobacter infections. This resistance occurs through a broad range of mechanisms, including antibiotic-hydrolyzing enzymes, efflux pump overexpression, decreased membrane permeability, and mutations in antibiotic targets. Acinetobacter shows a remarkable capacity to sustain an MDR phenotype, making treatment even more challenging. Acinetobacter spp. have the ability to hydrolyze β-lactams through the four different classes (A to D) of Ambler enzymes, produce aminoglycoside-modifying enzymes (AMEs), expel several antibiotics via efflux pumps, alter carbapenem and aztreonam access via porin mutations, and alter key antibiotic targets like penicillin-binding proteins, DNA gyrase, and lipopolysaccharide [15].

Enzymatic Inactivation

β-Lactamases and AME: Bacteria produce β-lactamases, which are enzymes that confer resistance to commonly used β-lactam antibiotics such as carbapenems, cephalosporins, penicillins, and monobactams. The production of β-lactamases, especially carbapenemases, poses significant challenges in clinical settings due to their potential to hydrolyze and inactivate carbapenems, which are often considered key antibiotics for the therapy of severe and MDR bacterial infections. AMEs inactivate aminoglycoside antibiotics by phosphorylation, adenylation, or acetylation, preventing them from binding to their bacterial ribosomal target and thereby eliminating their bactericidal activity [17].

Target Modification

*A. baumannii *employs target modification, a crucial resistance strategy that includes altering the antibiotic binding site to render the antibiotic ineffective. Through spontaneous or induced genetic changes and horizontal gene transfer, this process primarily affects β-lactams, aminoglycosides, and colistin [17].

Reduced Permeability and Active Efflux

One of the main antibiotic resistance mechanisms in *A. baumannii* is reduced permeability, which predominantly reduces the effectiveness of hydrophilic antibiotics, including tigecycline, aminoglycosides, and β-lactams. In fact, changes in the bacterial outer membrane prevent antibiotics from entering the bacterial cell, thereby lowering intracellular antibiotic concentrations and efficacy [17].

Biofilm-Associated Resistance

Biofilms are complex, well-organized communities of bacteria and/or fungi that are embedded in an extracellular polymeric matrix made of proteins, lipids, polysaccharides, and nucleic acids. The protective environment of the matrix enhances bacterial survival against the host immune system and antibiotic therapy [17].

Stenotrophomonas maltophilia: Treatment of infections caused by SM is notoriously challenging due to a range of intrinsic and acquired resistance mechanisms. Most β-lactams, fluoroquinolones, tetracycline derivatives (apart from doxycycline, minocycline, or tigecycline), chloramphenicol, all aminoglycosides (including kanamycin, tobramycin, amikacin, and neomycin), and trimethoprim (TMP) are among the many antibiotics to which this bacterium is intrinsically resistant [20].

Intrinsic Resistance

Overexpression or mutation of multidrug resistance efflux pumps, decreased membrane permeability, the chromosomally encoded Smqnr gene (which shields gyrase and topoisomerase IV from quinolones), and the production of β-lactamases, carbapenemases, and AMEs are among the major mechanisms of antibiotic resistance in SM. The production of two chromosomally encoded extracellular β-lactamases, blaL1 and blaL2, is the primary source of significant resistance to carbapenems in SM. This may explain the therapeutic advantage of ceftazidime-avibactam when used in combination with aztreonam [20].

Acquired Resistance

SM may acquire resistance to cefiderocol by various genetic mechanisms, including changes in the tonB gene or the smeT promoter. SM resistance to TMP-sulfamethoxazole has also been linked to the presence or acquisition of the sul1, sul2, and dfrA genes that exist in both chromosomal and plasmid. These genes are commonly linked with class 1 integrons, which facilitate gene mobility and may carry multiple resistance genes, contributing to multidrug resistance [20].

Burkholderia species** **(efflux pumps and drug resistance in *B. pseudomallei*): Efflux pump activity is the primary resistance mechanism affecting many antibiotic classes, according to a recent assessment of antibiotic resistance in *B. pseudomallei*. AmrAB-OprA, BpeAB-OprB, and BpeEF-OprC are the three resistance-nodulation-cell division-type drug efflux pumps identified to date [34].

AmrAB-OprA

In *B. pseudomallei*, this mechanism contributes to the high intrinsic resistance to macrolides and aminoglycosides [34].

BpeAB-OprB

In wild-type strains, its expression is minimal. BpeR controls the expression of BpeAB-OprB, and bpeR mutants have low levels of resistance to macrolides, fluoroquinolones, tetracyclines, and chloramphenicol [34].

BpeEF-OprC

Only regulatory mutants of *B. pseudomallei* express this pump; wild-type strains do not. For example, it is constitutively produced in bpeT mutations that occur spontaneously. Expression of BpeEF-OprC confers high-level resistance to TMP, fluoroquinolones, tetracyclines, and chloramphenicol. This mechanism contributes to the widespread resistance to TMP found in environmental and clinical *B. pseudomallei* isolates [34].

## Conclusions

Previously regarded as contaminants in hospital environments, NFGNB are now becoming a significant source of infection in intensive care units. This raises serious concerns due to the rapid development of resistance in NFGNB, even to new antimicrobial agents, which results in longer hospital stays, increased medical expenses, and higher morbidity and mortality rates. For effective patient care and to prevent the development of multidrug resistance, hospitals must properly screen for NFGNB, accurately identify them, and regularly evaluate their antibiotic susceptibility profiles. Antimicrobial stewardship policies and comprehensive microbiological surveillance systems must be rapidly implemented in hospitals, as evidenced by the rising rate of isolation of these resistant organisms. Furthermore, since these organisms have a high capacity to survive in a hospital setting, effective sterilization and infection control measures should be implemented. These include hand hygiene, the use of personal protective equipment, environmental sanitation, and the sterilization and disinfection of instruments and devices used in patient care, along with proper biomedical waste management.
